# Understanding Chinese Consumers’ Livestreaming Impulsive Buying: An Stimulus-Organism-Response Perspective and the Mediating Role of Emotions and Zhong Yong Tendency

**DOI:** 10.3389/fpsyg.2022.881294

**Published:** 2022-07-06

**Authors:** Hongli Gao, Xinzhi Chen, Hongling Gao, Bin Yu

**Affiliations:** ^1^School of Economics, Zhejiang University of Technology, Hangzhou, China; ^2^School of Economics and Management, Zhejiang University of Science and Technology, Hangzhou, China

**Keywords:** impulsive buying, SOR theory, Zhong Yong tendency, livestreaming, atmospheric cues

## Abstract

We built a livestreaming impulsive buying model based on stimulus-organism-response (SOR) theory, and we explored the impact of atmospheric cues (ACELS) and sales promotion (SPELS) on impulsive buying (IBI) based on emotions (EOC) and Zhong Yong tendency (ZYT) of online consumers. Combined with holistic orientation, perspective integration, and harmony maintenance, ZYT is a cognitive process involving individual events. We gathered 478 samples using a questionnaire to test the proposed research model. The empirical findings show that as the stimuli in the livestreaming environment, ACELS and SPELS during livestreaming greatly boost EOC while significantly constraining consumers’ ZYT. Among online consumers, positive EOC promotes IBI, whereas ZYT dampens it. In addition, EOC and ZYT mediate the relationship between stimulus factors and response factors in parallel, resulting in four model mediation paths. By incorporating the SOR model, this study provides theoretical underpinnings for the role of cognitive processing in impulsive purchases, as well as useful guidance for e-commerce platforms and streamers to effectively understand Chinese consumers’ purchase behavior, which benefits the development of effective promotion strategies and the creation of powerful marketing tools.

## Introduction

Livestreaming e-commerce delivers unique value and has endogenous growth potential because it (1) serves as a professional shopping guide for consumers and (2) saves e-commerce businesses from having to pay customer acquisition costs. During the COVID-19 pandemic in 2020, livestreaming became a critical driving force for economic development amidst a social and economic depression, especially in the Chinese market. According to the Chinese Statistical Report on Internet Development (CNNIC, 2020), users who purchase goods through livestreaming account for 66.2% of China’s 388 million livestreaming e-commerce users. Thus, for multinational enterprises and foreign brands, an in-depth understanding of Chinese consumers’ purchase behavior in the livestreaming environment is key to exploiting the local market (Cui and Liu, 2000, 2001).

Impulsive buying is widespread in conventional offline retail businesses, comprising up to 60% of supermarket sales and 80% of luxury goods sales (Zhang et al., 2010). The economic relevance of impulsive purchases is well documented in the retail sector (Verplanken and Sato, 2011), and Internet shoppers exhibit a higher level of impulsiveness than non-Internet shoppers (Greenfeld and Sutker, 1999; Brashear et al., 2009). Owing to the rapid advancement of technology—which allows for virtually instant gratification through immediate access to goods and services—impulsive consumption on the Internet has become pervasive (Baumeister, 2002; Vohs and Faber, 2007), occurring in about 40% of all online expenditures (Verhagen and Dolen, 2011). In academia, impulsive buying is defined as an unexpected purchase activity influenced by stimuli in the shopping environment, with three typical features: unplanned, illogical, and immediate (Rook, 1987; Lim et al., 2016). Although researchers believe that consumers’ cognitive evaluation of impulsive buying behavior may be the chief factor in determining impulsive purchases (Rook and Fisher, 1995; Dholakia, 2000; Omar and Kent, 2001), it remains unclear how cognitive processes work and how other psychological factors are involved in impulsive buying.

A thorough understanding of consumer traits and appropriate market segmentation is crucial for a multinational company’s success (Brashear et al., 2009). Given the uniqueness of Chinese culture, coupled with the institutional environment and historical reasons, differences in Chinese consumer behavior and marketing localization cannot be ignored. As a connotation of Chinese Confucian philosophy, Zhong Yong tendency (ZYT) occurs when we consider an issue from multiple angles and give thorough attention to different views that lead to decisions for both oneself and the general good (Wu and Lin, 2005). Chinese people have a larger predisposition to engage in ZYT when attempting to make sense of the world and then make judgments and take action (Peng and Nisbett, 1999). Wu and Lin (2005) pointed out that to capture the characteristics of ZYT reflected in Chinese psychology and behavior, we should start with specific situations, viewing ZYT as a cognitive process of individual events. The ZYT scale (Wu and Lin, 2005) has been utilized in a growing number of studies to examine the relationship between ZYT and conduct. For example, Ting-Yun and Cheng-Ta (2014) explored the relationship between people’s ZYT and their perceptual processing capacity. Wang et al. (2013) investigated how ZYT is linked to the qualities of banner ad-watching behavior. Although the link between ZYT and human behavior has been widely investigated, little is known about how people’s impulsive purchases are related to their ZYT in specific circumstances (i.e., in e-commerce livestreaming). We focused on ZYT to establish how it influences the formation of impulsive buying behavior, extending the conceptual domain of cross-cultural consumer research to include ZYT, and developing measures and manipulations of ZYT that can be readily used by consumer behavior researchers in the livestreaming environment.

The stimulus-organism-response (SOR) model shows how a stimulus can provoke an organism to engage in internal processes to prepare for the ultimate response. The literature related to purchase behavior in the livestreaming environment usually pays attention to stimuli and responses, but the internal mechanism of impulse buying remains unclear. According to Guo et al. (2021), livestreaming features greatly boost consumers’ overall perceived value and purchase intention. Lu and Chen (2021) found that livestreaming hosts’ physical attributes, conveyed through vicarious product trials and values shared *via* instant engagement, enable consumers with similar physical traits and values to reduce product uncertainty and cultivate trust. Sun et al. (2019) confirmed that IT affordability can affect customers’ purchase intention in livestreaming. Although impulsive buying is a rapid, spontaneous, and emotional phenomenon, internal processes remain significant (Huang, 2016). For example, Rook and Fisher (1995) considered impulsiveness as a cognitive construct and found a link between impulsiveness and impulsive behavior. ZYT describes the internal mechanism of consumers who seek the best state appropriately with a view of the whole situation and a mentality of self-control when faced with an external stimulus. Following the SOR framework, we explored an impact mechanism to suggest that online consumers’ ZYT and EOC are associated with impulsive buying in the livestreaming environment. This study also stresses the stimulus role of sales promotion and atmospheric cues, which influence customers’ impulsive buying intention (IBI) through livestreaming engagement.

## Literature Review

### Zhong Yong

Zhong Yong (the Doctrine of the Mean), the embodiment of the essence of Chinese Confucian philosophy, has an integrated definition and rich connotations. “Zhong” (中) means considering things carefully from different standpoints and integrating exterior and interior factors. One’s affect, cognition, and behavior are always experienced and expressed in moderation (Lin et al., 2020), which underlines appropriateness versus excessiveness, emotion, and conduct (Ji et al., 2010). “Yong” (庸) means “the way,” highlighting fixed principles and unchangeable routines (Li et al., 2019). The preservation of harmony, or the union of people and nature, is a core value of Zhong Yong (Zhou et al., 2019b). Wu and Lin (2005) found that when making decisions, people with high Zhong Yong prioritize self-control, avoid fluctuations in ephemeral feelings, and modify their behavior according to their surroundings. Zhong Yong is not only a distinctive, vital philosophical concept in traditional Chinese culture but also a way of thinking that is used habitually by Chinese people (Yang, 2010). Tang et al. (2020) found that low levels of Zhong Yong are better for employee creativity in unpredictable situations as they cause overemphasis on compromise and giving in when times are uncertain. In addition, Zhong Yong plays an important role in Chinese consumers’ decision-making process (Aimee et al., 2020). Sheng et al. (2019) revealed that Zhong Yong thinking is positively associated with green purchase intention among Chinese consumers based on three dimensions of consumer lifestyle (leadership, cost consciousness, and development consciousness). In addition, by indicating the moderating role of Zhong Yong thinking, Wang et al. (2021) demonstrated that people make an effective shift from an emotion-based hot-processing system to a cognitive-based cold-processing system to acquire self-control in their behavior in the face of major occurrences such as the COVID-19 pandemic, which results in impulsive buying behavior.

Numerous scholars have adopted Zhong Yong as a decision-making style and a type of characteristic, introducing Zhong Yong thinking as a moderator (Tang et al., 2020; Wang et al., 2021). However, according to Rokeach (1968), all interpretations of values are made up of three parts: a cognitive component that represents information, an affective component that can elicit feelings, and a behavioral component that is engaged when action is necessary. As a highly practical thinking style, Zhong Yong has a behavioral dimension that cannot be overlooked because individuals subjectively encode and create varied interpretations of the events that they face (Chen et al., 2020). On the one hand, Zhong Yong is a long-term, consistent personality trait and is seen as a cultural value; on the other hand, it can be modified by external circumstances since it is a blend of many ways of thinking that can be altered depending on the scenario (Zhou et al., 2019a). Based on this, researchers have begun to verify the mediating role of Zhong Yong in many circumstances. Hou et al. (2020) investigated the mediating effects of Zhong Yong on the relationship between parenting style and emotional distress among college students (i.e., symptoms of depression and anxiety). Lin et al. (2020) verified that honesty-humility is positively correlated with dispositional awe, and this relationship is mediated by Zhong Yong thinking. According to Ting-Yun and Cheng-Ta (2014), individual differences in perceptual processing capacity are predicted by Zhong Yong, indicating that culture can influence cognitive processing style.

The Zhong Yong thinking scales (Chiu, 2000; Wu and Lin, 2005; Huang et al., 2012) have been utilized in a growing number of studies to show a link between ZYT and human conduct in a specific circumstance. The findings of them all pointed out that high Zhong Yong thinkers use a more broad and flexible cognitive processing style when engaging in the outside world. Using the Zhong Yong thinking Scale (Huang et al., 2012; Wang et al., 2013) investigated how ZYT affects banner ad viewing and evaluation. The result showed that high Zhong Yong thinkers were more sensitive to banner complexity and adopted a more global strategy to combine information from all regions of the advertisements compared to low Zhong Yong thinkers. Also, in the experiments of Ting-Yun and Cheng-Ta (2014), participants completed a Zhong Yong Thinking Scale (Wu and Lin, 2005) and performed a redundant-target detection task. The relationship between an individual’s ZYT and perceptual processing capacity was explored, which revealed that users who exhibited a greater extent of Zhong Yong thinking processed information more effectively in an integrated manner. As a result, the study deeply explored ZYT and considered it as an intermediary variable between marketing stimulation and consumption behavior in the scenario of livestreaming. We refer to the dominant framework of Wu and Lin (2005), who investigated Zhong Yong from the perspective of decision-making style in three dimensions: holistic orientation, perspective integration, and harmony maintenance.

First, holistic orientation expresses an inclination to deliberate on a situation from a global standpoint and to consider all constituent details when making decisions (Chou et al., 2014). This is reflected in consumers’ decision-making processes, which involve long-term and short-term benefits and costs. Second, perspective integration requires individuals to incorporate perspectives on all sides, which facilitates reasonable resolution when people adopt compromising approaches to reconciling conflicting forces (Ji et al., 2010). As a compromise, perspective integration results in self-reflection and self-control under an external stimulus. Third, the ultimate goal of holistic orientation and perspective integration is to maintain harmony (Wu and Lin, 2005), which is an ideal state for relationships and a way to deal with conflict without having to resort to severe measures (Qu et al., 2018). When faced with an external stimulus, people with ZYT harmoniously attain their goals and make fair choices after considering internal and external factors.

### Impulsive Buying

The concept of impulsive buying was first proposed by DuPont (the DuPont Consumer Buying Habit Studies, 1945–1965), who found that impulsive buying is the difference between real purchase behavior and a purchase plan. Rook (1987) defined impulsive buying as “buying something immediately when experiencing a strong impulse,” from the perspective of consumer psychology and behavior. With the rapid development of internet technology and e-commerce, academia has begun to study impulsive buying in online scenarios. Eroglu et al. (2001) asserted that online shopping platforms rely on technical support, which provides more favorable and convenient conditions for impulsive shopping, such as convenient commodity information searches while shopping (Verhagen and Dolen, 2009), fewer time–space constraints (Eroglu et al., 2001) and convenient online payment options (Kwon and Armstrong, 2002). In addition, promotion methods such as the lottery (Larose, 2006) and users’ increasing dependence on online purchases (Adelaar et al., 2003) have made impulsive buying more common. However, some scholars maintain that traditional brick-and-mortar approaches have advantages in stimulating impulsive buying (Madhavaram and Laverie, 2004).

Livestreaming e-commerce combines the high convenience of traditional online purchases with strong atmospheric cues and interactions in an offline purchase, which has the characteristics of authenticity, immersion, community, and timely interactions. The exterior design of live platforms (Gong et al., 2020), atmospheric features (Gong et al., 2019), and interaction text (Fei et al., 2020) have a significant impact on consumers’ impulsive purchases. Combined with atmospheric cues and different promotion methods *via* livestreaming, we established a corresponding research model and examined the influence path and mechanism of atmospheric cues and sales promotion on IBI through empirical test results.

Previous studies on cross-cultural differences in impulsive buying behaviors indicate that Western consumers exhibit more impulsive buying behaviors than Eastern consumers. Zhang et al. (2010) affirmed that beyond related cultural dimensions, the power distance belief (PDB) proposed by Hofstede (1984) has a great impact on impulsive buying. In Eastern societies with high-power distance beliefs, consumers have a stronger intention toward obedience and self-control (Zhang et al., 2010). While Westerners value the present more than the future, they prioritize immediate gratification over restraint or delayed gratification (Chen et al., 2005) and are thus more likely to make an impulsive purchase. Markus and Kitayama (1991) also proposed that people’s impulsive buying behavior is influenced by self-construal (i.e., seeing oneself as tied to other people or not). Consumers with independent self-construal regard themselves as independent and autonomous, placing high importance on individuality and accomplishments, while consumers with interdependent self-construal view themselves as part of a broader group valuing connectivity, conformity, and group harmony, placing a high priority on safety and security (Zhang and Shrum, 2009). Although cultural differences can affect customer behavior, most existing research on consumers’ impulsive buying behavior is based on Western theoretical systems, ignoring the unique cultural traits of local circumstances in China.

### Applying SOR Theory

Based on environmental psychology, the SOR model was first proposed by Mehrabian and Russell (1974), which indicates that stimulating cues (i.e., stimuli) from the environment trigger one’s internal evaluation (i.e., organism), which leads to an approach-and-avoidance reaction (i.e., response). Stimuli are triggers that elicit a response from customers and primarily include marketing stimuli such as promotions (Longdong and Pangemanan, 2015; Hasim et al., 2018), pricing (Xu and Huang, 2014; Zou, 2018), and scarcity (Wu et al., 2020b); website stimuli such as media format (Adelaar et al., 2003), payment features (Dutta et al., 2003), and visual appeal (Liu et al., 2013); and situational stimuli (Park et al., 2012).

**Figure 1:**
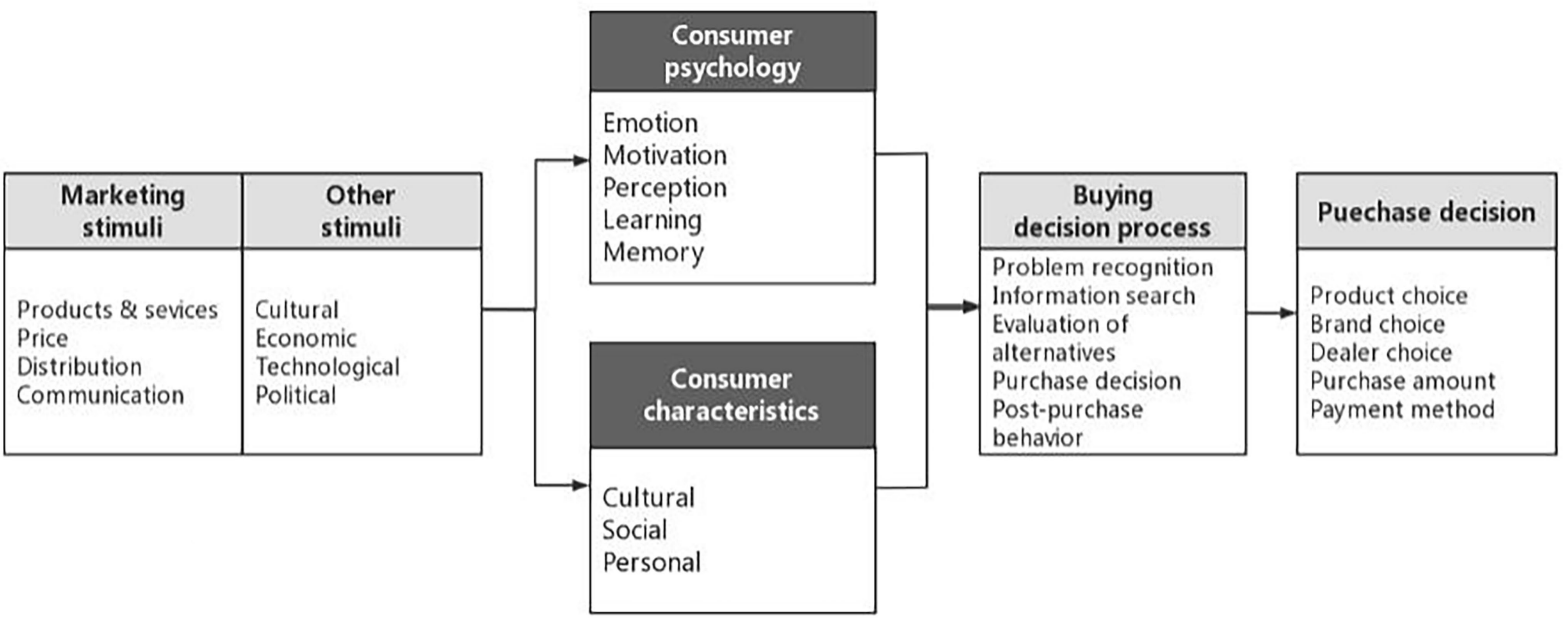
Model of consumer behavior based on traditional stimulus-organism-response (SOR) theory. Source: Kotler and Keller (2007).

Organisms represent consumers’ internal processes and mental states from the stimulus to the response, including cognitive and affective intermediary processes. The cognitive reaction encompasses everything that goes on in people’s minds in terms of knowledge acquisition, processing, retention, and retrieval, including attitudes, beliefs, attention, comprehension, memory, and knowledge (Eroglu et al., 2001). Shoppers’ affective reactions can be conceptualized as emotional responses that take place when people engage with their surroundings (Sun and Zhang, 2006), such as arousal (Lin and Lo, 2015), pleasure (Khalifa and Shen, 2007), enjoyment (Wu and Ye, 2013), flow experience (Hsu et al., 2012), and dominance (Adelaar et al., 2003). On the theoretical basis of the SOR model, Kotler and Keller (2007) developed a consumer behavior model (Figure 1), arguing that external stimuli affect consumers’ psychology and characteristics, both of which play a critical role in intervening in the relationship between a stimulus and an individual’s response. In this study, the emotional factor represents consumers’ psychological state in the face of external incentives. Meanwhile, ZYT fundamentally determines wants and behavior as a cultural factor, as well as choices and desires over time as a personal factor (i.e., core value). Based on this, we examined EOC and ZYT as affective and cognitive constructs, respectively, which comprise the organism of the SOR model.

As an outcome variable, a response denotes one’s approach or avoidance reaction following psychological changes, including psychological and behavioral responses (Donovan and Rossiter, 1982; Sherman et al., 1997). Marketing and environmental stimuli penetrate consumers’ consciousness, and a set of psychological processes combine with certain consumer traits to produce decision-making processes and purchase choices.

After reviewing the literature, it was noted that marketing research focuses on consumer behavior in physical stores and traditional online environments. However, over time, an increasing number of people have turned to livestreaming e-commerce for shopping. New types of stimulation are generated by new retail environments. The impact of new stimulation on consumer behavior, as well as the extent to which the mechanism and behavior boundary conditions of previous consumption conduct can migrate to a new environment, have become a focus of research. Rooted in SOR theory, Yang et al. (2021) built a model of consumers’ purchase intention for fresh agricultural products under a livestreaming situation, introducing perceived trust and perceived risk as mediating variables. Zuo and Xiao (2021) explored the influence of cues from the livestreaming shopping environment on consumers’ impulsive buying behavior and the mediating roles of cognitive reactions (perceived usefulness) and affective reactions (perceived enjoyment).

Thus, we refer to the past literature, and we selected this mature theoretical framework to build the theoretical model. In the context of live e-commerce, we chose SPELS and ACELS as the stimuli, ZYT, and the EOC as the organism, and IBI as the response. Based on this model, we explored the internal relationships between diverse variables and the impact mechanism of impulsive buying to expand the application scope of SOR theory to the livestreaming environment. In particular, we introduced the variable of ZYT into the SOR model to depict typical Chinese psychology in the context of livestreaming.

## Theoretical Development and Hypotheses

### Stimulus Factors of Livestreaming IBI

#### The Effect of SEPLS

Sales promotion is an important marketing method that affects consumer purchase decisions. It is usually designed around a time limit and material incentives (Lu and Qin, 2013). In the context of livestreaming it refers to things such as “flash sales,” coupons for a limited time, and an exclusive price for live broadcasts; it gives consumers a certain kind of discount within a given period or some pure material incentive like lucky draws. The time limit is visible and updated promptly, which imposes time pressure on the viewer (Wu et al., 2021), influencing inner cognition and affect.

According to the pleasure-dominance-arousal (PDA) model proposed by Mehrabian and Russell (1974), emotion reaction comprises three dimensions: arousal, pleasure, and dominance. We focused on the effects of arousal and pleasure on impulsive buying behavior, as they are sufficient to indicate the emotional responses and states brought about by external stimuli (Russell and Pratt, 1980). Pleasure gauges the degree of happiness and satisfaction, while arousal measures the degree of stimulation and excitement when consumers are stimulated in a livestreaming environment.

Consumers’ pleasure and arousal may be affected by online promotion strategies, price discounts, and their subjective disposable time. Finucane et al. (2000) discovered that people under time constraints are more likely to rely on affect, which is accompanied by increased arousal levels. Maule et al. (2000) found that time pressure positively affects an individual’s arousal. Consequently, the sensation of time pressure is created by a short decision-making window and arouses viewers during livestreaming. Furthermore, the influence of time scarcity on arousal may be exacerbated by visualizing the time limit (Wu et al., 2021). In addition, according to the transaction utility theory in commodity pricing developed by Thaler (1983), price discounts give consumers the perception of lower expenses, whereas non-price promotions give consumers the impression of higher benefits or utility. Consumers’ transaction utility is improved owing to the larger potential benefits provided by promotions; thus, purchases are more pleasurable for consumers.

According to Inbar et al. (2010), decision-makers are more inclined to use heuristic thinking to make quick choices under time constraints. De Dreu (2003) showed that time pressure reduces individuals’ (self-reported) motivation to absorb information and increases their reliance on inadequate decision heuristics. As a result, during livestreaming promotion with high time constraints, ZYT, used to think comprehensively and integrally, may be inhibited. Consumers will struggle to use ZYT to develop a thorough view of a product as they lack the time to gather comments and combine the opinions of others and themselves. In addition, due to higher discounts, viewers’ utility of obtaining timely satisfaction may exceed that of delaying a purchase, so they are less likely to estimate the rationality of purchase decisions from a long-term perspective and to violate ZYT. Thus, we formulated the following hypotheses:

*H1*: SPELS dampens ZYT.

*H2*: SPELS promotes EOC.

#### The Effect of ACELS

Atmospheric cues emerge in a shopping environment that stimulates consumers’ interest in shopping (Demangeot and Broderick, 2010). E-commerce livestreaming is a unique setting in which traditional offline and online purchases are lacking. Compared to web-based e-commerce, livestreaming can foster more authenticity and interactivity during online shopping, causing more customers to make impulsive purchase decisions (Sun et al., 2019; Ming et al., 2021). Unlike traditional establishments, e-commerce livestreaming allows for “multi-to-multi” communication, as opposed to “one-to-one.” Liu et al. (2020) found that the interactive, authentic, entertaining, and visual features of e-commerce livestreaming affect consumers’ purchase behavior by activating their emotional or cognitive responses. Hence, we chose three typical features of atmospheric cues in e-commerce livestreaming: the “interactive atmosphere,” the “entertainment atmosphere,” and the “safety atmosphere.” The “interactive atmosphere” refers to two-way information exchanges between the viewer, the anchor, and the audience *via* the (bullet) screen. The “entertainment atmosphere” entails a type of setting that can make viewers feel satisfied, such as the anchor’s amusing explanation or interesting content on the (bullet) screen. The “safety atmosphere” denotes the degree to which viewers trust a live description to match a genuine one.

E-commerce livestreaming, as a sales platform with tremendous engagement and entertainment features, can stimulate audiences’ emotional reactions in the following ways. First, the bullet screen will enhance consumers’ entertainment and flow experiences (Chen and Lin, 2018), immersing them in the diversity of entertainment. Second, highly entertaining livestreaming can help consumers to experience an excited and pleasant mental state while shopping (Ma and Mei, 2018). Third, the more trustworthy a website is, the more comfortable consumers will be (Van Der Heijden and Verhagen, 2004), and higher perceived security brought about by the real 3D display of live broadcasts enhances viewers’ feelings of pleasure.

The interactivity and entertainment atmosphere created by interacting with the anchor and other viewers through (bullet) screens make viewers fall into a flow state, with a focus on watching phenomena live and posting comments (Li and Peng, 2021). Individuals’ concerns regarding external assessments decrease when entering a flow state (Sinnett et al., 2020), thus failing to activate ZYT, which leaves them unable to think holistically and integrally, causing them to fail to judge the authenticity of external information and the rationality of their behavior. By reducing the perceived risk of making a purchase, the security atmosphere prevents viewers from engaging in further consideration, namely by restraining ZYT. As such, we formulated the following hypotheses:

*H3*: ACELS restrains ZYT.

*H4*: ACELS invokes EOC.

### The Organism of Livestreaming IBI

#### The Effect of ZYT on IBI

Moser et al. (2019) indicated that encouraging deliberation and avoidance can help customers avoid making impulsive purchases. According to Vohs and Faber (2007), the depletion of self-regulatory resources leads to impulsive buying behavior. Individuals who experience ZYT tend to have a moderate attitude and monitor their behavior constantly, restraining their desires and inhibiting impulsive purchase intention due to self-control (Vohs and Faber, 2007). Furthermore, those with ZYT tend to interpret information holistically rather than focusing on short-term temptations. They recognize and externalize the long-term cost of impulsive buying, reducing impulse buying intention by balancing pros and cons. Thus, we formulated the following hypothesis:

*H5*: ZYT limits IBI.

#### The Effect of EOC on IBI

Youn and Faber (2000) asserted that emotions serve as potential internal stimuli that trigger impulsive buying. Consumers experience complex emotional processes in impulsive buying (Rook, 1987), including strong impulses before shopping, as well as excitement and pleasure during shopping. In academia, it is widely believed that a substantial portion of impulsive buying by customers is due to their favorable emotional reactions (Hausman and Siekpe, 2009; Wu et al., 2020a). Jeon (1990) and Chang et al. (2012) confirmed that emotional pleasure and arousal both directly affect impulsive buying behavior. When consumers experience high levels of arousal and pleasure, they maintain a positive mood, exaggerate their requirements and economic strength, and diminish the intensity of rational thought, which easily results in impulsive buying. Accordingly, consumers’ positive affective reactions—that is, a high degree of pleasure and arousal—have a positive impact on IBI. Thus, we developed the following hypothesis:

*H6*: EOC promotes IBI.

#### The Effect of EOC on ZYT

From the sociocultural standpoint of emotions, Mancuso and Sarbin (1998) defined emotions as a comprehensive system whose generation, maintenance, and recovery are influenced by circumstances and the cognition process. According to Gross (2010), emotion regulation methods operate at the cognitive level. ZYT may have an impact on emotions because it encourages people to look at things from many angles and to avoid experiencing or expressing intense sensations (Ji et al., 2010). The global and flexible cognitive processing style of people with ZYT has been identified (Wang et al., 2013). Yang (2008) pointed out that ZYT is involved in two stages of emotional expression. When emotions are not exhibited, ZYT stops them from forming because people focus more on careful consideration before generating a mood, as well as calming down sentiments and disturbances; this is rarely addressed in Western emotional psychology. The other stage entails self-control and self-restraint once emotions have been formed, which can have a favorable psychological impact on people.

Hence, the impact of ZYT on emotion regulation is multi-dimensional, including the thinking process at the metacognitive level (Ji et al., 2010) and the use of specific cognitive methods to integrate situational information and event clues. This is consistent with the view that ZYT is not only a value but also a method and practice in philosophical speculation. As such, we posited that the ZYT would influence emotional control.

*H7*: ZYT limits EOC.

### The Mediating Role of EOC and ZYT

Consumers’ impulse to make a purchase is characterized by high affective activation, which requires minimal or little cognitive effort to develop (Weinberg and Gottwald, 1982; Ning Shen and Khalifa, 2012). According to the “affective-cognitive” model, proposed by Shiv and Fedorikhin (2002), consumers’ cognitive load, decision-making time, external stimulus form, and other aspects alter available processing resources, increasing the role of affect in decision-making and decreasing the contemplation of repercussions of choices. People are more likely to make irrational purchase decisions when their cognitive load is high (Liu, 2014). When encountering encouraging promotional information in the form of time constraints, material incentives, and highly interactive and entertaining ACELS, consumers are mentally preoccupied and must make choices quickly, so they are more likely to make decisions based on EOC rather than ZYT, which leads to IBI. Thus, we posited that sales promotion and atmospheric cues would promote impulse buying, inducing positive EOC and suppressing ZYT.

EOC can mediate between marketing stimuli (i.e., ACELS and SPELS) and IBI, serving as a psychological process triggered by the shopping environment. Wu et al. (2021) hypothesized that limited-time scarcity could increase customers’ perceived arousal, leading to online impulsive buying based on the competitive arousal model (Ku et al., 2005). The sales promotion of livestreaming will place consumers under time constraints and arouse them. They will be enticed by tangible rewards such as a lucky draw, thereby succumbing to the anchor’s persuasion and having an irresistible IBI. Chang et al. (2011) found that the characteristics of the retail environment influence impulse buying through consumers’ positive emotional responses. Interactivity and entertainment atmospheres, such as anchors’ interaction orientation, will have a positive effect on viewers’ immersion (Liao et al., 2022), causing people to lose track of time, disregard their surroundings, and experience intense pleasure and arousal (Van Noort et al., 2012). Hence, consumers are readily affected by the persuasion of anchors, leading to more impulsive buying behavior.

**Figure 2:**
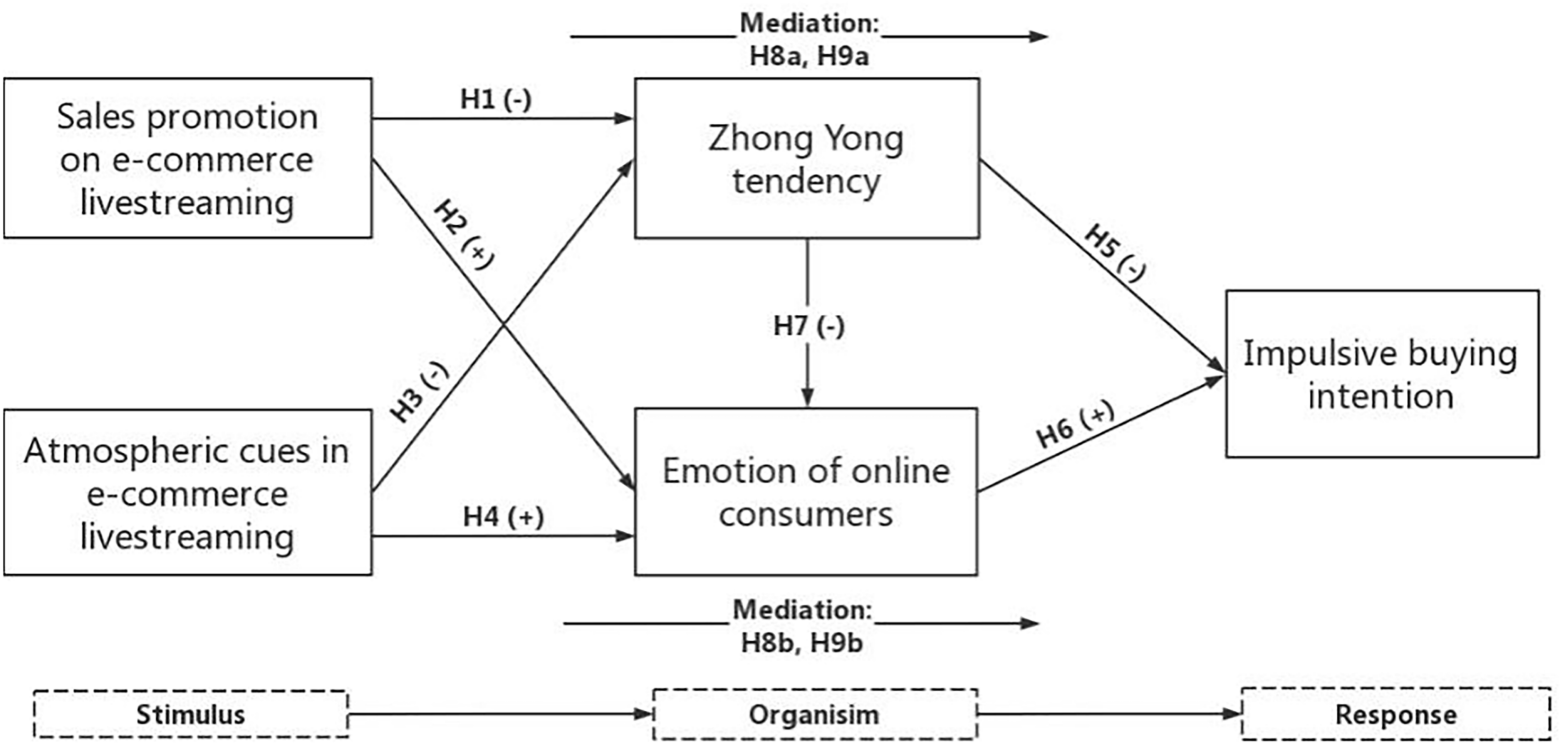
Research model.

Individuals under time constraints are prone to using an “affect heuristic” (Finucane et al., 2000) in which consumers can only observe the surface information of transactions and are more impulsive in their decision-making (Swain et al., 2006). According to Wu and Huan (2010), high time pressure makes it more difficult for customers to holistically analyze hazards, thus leading to impulsive purchases. In livestreaming, the time pressure imposed by visual and real-time updated time displays limits consumers’ cognitive resources, reducing the breadth and depth of their thinking and thereby inhibiting their ZYT, making it difficult for them to form a comprehensive, objective view of goods. Consequently, consumers are more likely to be swayed. As such, consumers are more likely to be influenced by sales promotions and purchases on a whim. The enthusiastic interactions between viewers and anchors cause consumers to become immersed in livestreaming, leading them to enter a flow state (Li and Peng, 2021) that prevents them from further evaluating a given product. By rendering a highly interactive and entertaining atmosphere, consumers’ IBI is fostered (Figure 2). Thus, we formulated the following hypotheses:

*H8a*: ACELS is positively related to IBI *via* the mediating effect of ZYT.

*H9a*: SPELS is positively related to IBI *via* the mediating effect of ZYT.

*H8b*: ACELS are positively related to IBI *via* the mediating effect of EOC.

*H9b*: SPELS is positively related to IBI *via* the mediating effect of EOC.

## Methodology

### Questionnaire Design and Data Collection

The initial stage of this study involved building the research model and questionnaire items. To obtain the validated scales employed in our research, we referred to several relevant documents and included some scale items in the original pool as candidate questionnaire items. In addition, we adapted some self-developed questions from classic literature to suit the livestreaming purchase environment. After gathering the candidate measurement items to improve the project, we held numerous personal interviews with young college students from a major university in Zhejiang Province. The iterative interview approach demonstrated that the scale entries accurately reflected the required phenomena, and essential ideas were not missing from the conceptual framework development domain.

The final questionnaire consisted of three parts. The first part explains the basic information of the questionnaire, including a description of the livestreaming promotion scene, IBI, and pertinent instructions from the Doctrine of Zhong Yong. Participants were asked to fill out the questionnaire according to their most recent live shopping experience. We used the second part to collect basic information on the participants, including their gender, age, monthly income, education level, and monthly disposable income. The third part encompasses the five variable scales involved in the research model, in which we measured 17 questions.

We collected questionnaires through the Wenjuanxing app, which is a widely used data collection app in China. We distributed 500 questionnaires, of which we collected 485. We utilized an attention check question to see if the participants were paying attention to the survey questions, which helped improve the quality of the acquired empirical data. After deleting those with completely identical answer options and those with less than 1 min of answer time, 478 valid questionnaires remained, with a response rate of 95.6%. The descriptive statistics of the survey samples are presented in Table 1; 47.3% of the respondents were male and 52.7% were female. In addition, 64.8% had a bachelor’s degree or above, and most were aged between 18 and 31 years, accounting for 44.1%.

**Table 1:** The demographic traits of the respondents (*N* = 478).

Variable	Category	Frequency	Percentage (%)
Gender	Male	226	47.3
	Female	252	52.7
Age (years)	Under 18	88	18.4
	18–31	211	44.1
	31–40	95	19.9
	41–50	84	17.6
Education level	High school and below	47	9.80
	Higher vocational school (including enrollment)	121	25.3
	Bachelor’s degree (including enrollment)	199	41.6
	Graduate degree or higher (including enrollment)	111	23.2
Monthly income (RMB)	Under 1,000	27	5.60
	1,000–3,000 (including 3,000)	196	41.0
	3,000–5,000 (including 5,000)	59	12.3
	500–7,000 (including 7,000)	60	12.6
	7,000–9,000 (including 9,000)	54	11.3
	Above 9,000	82	17.2

### Variable Measurement

To ensure the accuracy and practicability of the questionnaire, we based all measurement items on the previous maturity scale. For the present study, the items were adapted to fit the context of e-commerce livestreaming. We used a five-point Likert scale ranging from 1 (strongly disagree) to 5 (completely agree) to gauge all items. As it originated from traditional Chinese culture, we derived the measurement of Chinese ZYT from Confucian philosophy. ZYT was measured by the Zhong-Yong Thinking Style Scale, which was developed by Wu and Lin (2005) and Zhou and Yang (2021). The Zhong-Yong Thinking Style Scale consists of three factors: holistic orientation, perspective integration, and harmony maintenance. We took the EOC scale from Cheng et al. (2009) and Mehrabian and Russell (1974), including pleasure and arousal. We adapted the three measurement items of ACELS from Wang et al. (2022), Dmitri (2017), Chen and Lin (2018) and Wongkitrungrueng and Assarut (2020), including the interactivity, entertainment, and safety atmospheres. We obtained four items measuring SPELS from Chou (2019), Lin and Lin (2013), Duncan Herrington and Capella (1995) and Rook (1987), including two dimensions: time limits and material incentives. We took the items measuring IBI from Beatty and Ferrell (1998), Liu et al. (2013) and Dmitri (2017) (see Appendix for full measurement items of all the variables).

## Model Validation

### Measurement Model Tests

To assess the model’s fitness, we used Smart PLS 3.0 and SPSS 24 to examine its reliability, convergent validity, and discriminant validity. We performed reliability tests using Cronbach’s alpha, composite reliability (CR), and average variance extracted (AVE; Nunnally, 1978). According to the SPSS reliability test, the Cronbach’s alpha values for all constructs varied from 0.901 (EOC) to 0.944 (IBI), which was significantly greater than the recommended threshold of 0.70 (Hair et al., 2006). Internal consistency can be ensured by determining the composite reliability of constructs (Fornell and Larcker, 1981). The composite reliability values were all greater than 0.7 (Hair et al., 2017a,b), ranging from 0.911 (ACELS) to 0.934 (IBI). The Roh_A value for each construct was well above the recommended threshold (Dijkstra and Henseler, 2015) of 0.7, revealing values ranging from 0.872 (EOC) to 0.907 (ZYT). This shows that the internal consistency of the measurement items was good, and the reliability was acceptable.

We classified the validity tests into convergent and discriminant validity to ensure that the questionnaire was valid. For the convergent validity tests, we established the degree to which operationalization is similar to (i.e., converges on) other operationalizations, which should theoretically be similar. We used the AVE values to check for convergent validity. As seen in Table 2, the AVE of each latent variable is greater than 0.5 (Fornell and Larcker, 1981), indicating convergent validity. In addition, all items in the validity analysis had factor loadings >0.725, which exceeded the acceptable level of 0.70 (Hair et al., 2017c). For discriminant validity, we examined the degree to which the operationalization is not similar to (i.e., diverges from) other operationalizations, which theoretically should not be similar. We assessed the discriminant validity of the constructs using the method developed by Fornell and Larcker (1981) comparing the square root of each latent variable’s AVE with the correlation coefficient of each latent variable, based on the idea that a construct shares more variance with its associated indicators than any other construct. As presented in the cross-loading matrix in Table 3, each item’s correlation coefficient was larger than the correlation coefficient in other dimensions, exhibiting sufficient discriminant validity (Wynne, 1998). We confirmed the convergent and discriminant validity of all constructs of the proposed research model.

**Table 2:** Analytical results of the factor loading, mean, SD, Cronbach’s alpha, AVE, roh_A, and composite reliability.

Construct	Items	Factor loading	Mean	SD	*α*	CR	AVE	rho_A
ACELS	ACELS1	0.778^***^	3.021	0.964	0.921	0.911	0.674	0.878
	ACELS2	0.871^***^	3.238	1.392				
	ACELS3	0.878^***^	3.372	1.298				
	ACELS4	0.720^***^	3.441	1.690				
	ACELS5	0.848^***^	3.184	1.352				
EOC	EOC1	0.844^***^	2.952	1.025	0.901	0.912	0.723	0.872
	EOC2	0.834^***^	2.646	1.112				
	EOC3	0.823^***^	2.946	1.320				
	EOC4	0.898^***^	3.021	1.271				
IBI	IBI1	0.938^***^	2.607	1.780	0.944	0.934	0.780	0.904
	IBI2	0.762^***^	3.052	1.029				
	IBI3	0.918^***^	3.067	1.570				
	IBI4	0.904^***^	2.657	1.387				
SPELS	SPELS1	0.845^***^	2.854	1.153	0.934	0.923	0.750	0.889
	SPELS2	0.844^***^	2.579	0.738				
	SPELS3	0.889^***^	2.879	1.110				
	SPELS4	0.886^***^	3.132	1.260				
ZYT	ZYT1	0.882^***^	3.073	1.324	0.938	0.929	0.687	0.907
	ZYT2	0.920^***^	3.471	1.145				
	ZYT3	0.829^***^	3.199	1.269				
	ZYT4	0.769^***^	2.678	1.085				
	ZYT5	0.906^***^	3.437	1.215				
	ZYT6	0.725^***^	2.994	1.457				

*SD, standard deviation; *α*, Cronbach’s alpha; DV, dependent variable; AVE, average variance extracted; and CR, composite reliability*.

*** *Significant at *p* < 0.01*.

**Table 3:** Analytical results of discriminant validity (Fornell–Larcker criterion).

	ACELS	EOC	IBI	SPELS	ZYT
ACELS	0.821				
EOC	0.785	0.850			
IBI	0.790	0.773	0.883		
SPELS	0.813	0.800	0.845	0.866	
ZYT	−0.747	−0.690	−0.828	−0.785	0.829

*ACELS, atmospheric cues in e-commerce livestreaming; EOC, emotions of online consumers; IBI, impulsive buying intention; SPELS, sales promotion in e-commerce livestreaming; ZYT, Zhong Yong tendency. Diagonal elements are the square roots of the AVE of the construct*.

Next, we put all variables into exploratory factor analysis (EFA), tested the outcomes of non-rotational factor analysis, and determined the minimum number of factors necessary to explain the variation in the variables. If only one factor has special explanatory power, a serious common method deviation can be determined (Eby and Dobbins, 1997; Livingstone et al., 1997). We added a potential marker variable to the hypothetical model, which is theoretically unrelated, and calculated the correlation between the unrelated and study variables. The path coefficients were consistent with those of the original model, with a few differences. Thus, common method variance is not a serious problem in this research model.

### Structural Modeling Test

**Figure 3:**
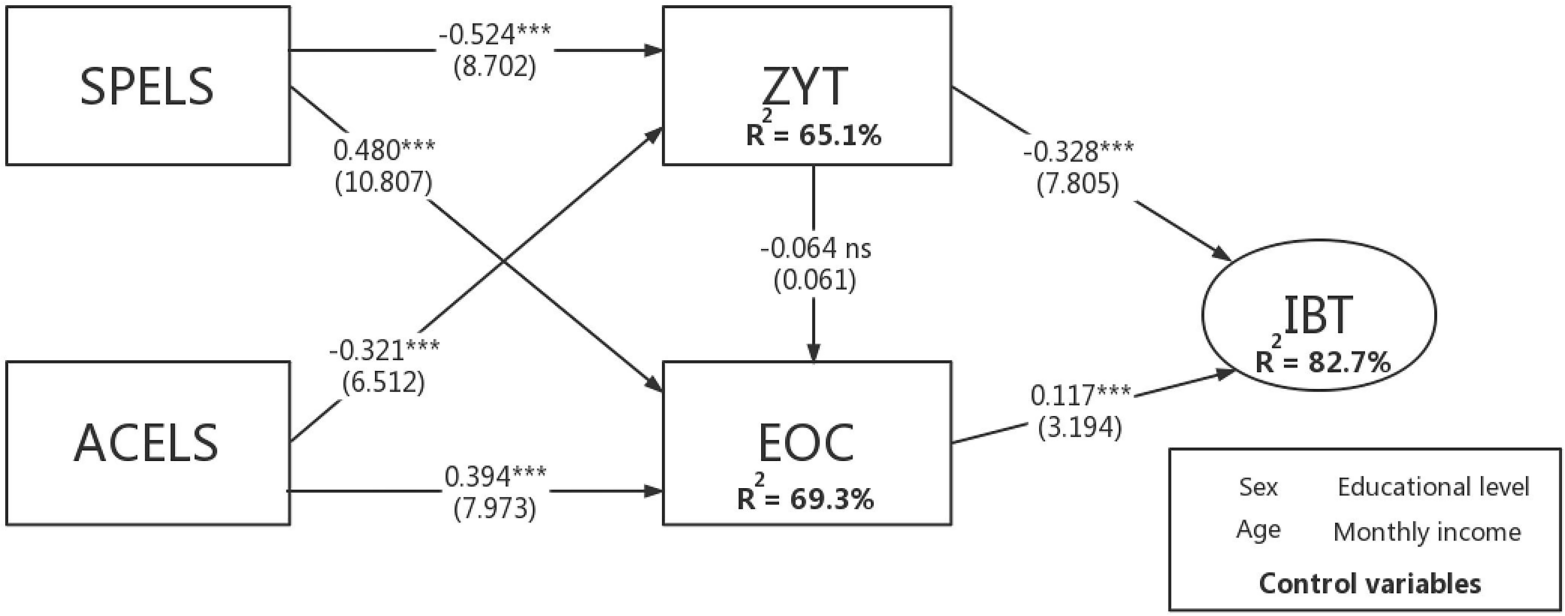
PLS results of the research model. ***, stands for the significance level of 0.001 and ns, refers to insignificance.

We utilized path analysis to examine the effects in the research model and to test the hypotheses (Hair et al., 2006; Salari et al., 2020). The results are presented in Figure 3. Based on our discussion and review of earlier studies, we employed a bootstrapping technique to obtain the t-statistic, determining the significance of the paths using 5,000 resamples (Peng and Lai, 2012; Hair et al., 2017b) and incorporating control variables (sex, age, education level, and monthly income) to apply ACELS, EOC, IBI, SPELS, and ZYT. Figure 3 depicts the path coefficients and explains the variances in the conceptual model employed in this investigation. We calculated the factor scores of the five key constructs using the means and SDs of the bootstrap samples from (partial least squares) PLS analysis.

For H1, a significant path coefficient of −0.524 (*t* = 8.702, *p* < 0.01) confirmed the negative effect of promotion on ZYT. This finding indicates that the promotion strength of livestreaming broadcasts significantly affects Chinese buyers’ ZYT. The significant path coefficient of 0.48 (*t* = 10.807, *p* < 0.01) demonstrates a positive relationship between SPELS and EOC, i.e., H2 is supported. In line with our proposed hypothesis, the PLS analysis provides strong support for H3 (*β* = −0.321, *t* = 6.512, *p* < 0.01) regarding the negative relationship between ACELS and ZYT. H4 predicted that the atmosphere of livestreaming would stimulate EOC. In this case, the hypothesis (*β* = 0.394, *t* = 7.973, *p* < 0.01) was supported. The influence of ZYT on IBI (i.e., H5) was confirmed by its significant path coefficient of −0.328 (*t* = 7.805, *p* < 0.01). This finding suggests that ZYT negatively affects the IBI of consumers in livestreaming promotion. According to H6, the direct link between EOC and IBI is positive. The outcomes also support this hypothesis (*β* = 0.117, *t* = 3.194, *p* < 0.01). However, contrary to our hypothesis, the effect of ZYT on EOC is not significant (*β* = −0.064, *t* = 0.061, *p* > 0.01); that is, H7 does not hold.

In addition to evaluating the significance of the path coefficients, we assessed the model’s predictive power by observing the coefficient of the endogenous structure to determine the efficiency value (Hair et al., 2014). We computed the *R*^2^ of related constructs; the results are shown in Figure 3, with 65.1% of the variance in ZYT and 69.3% of the variance in EOC. In addition, both ZYT and EOC explain 82.7% of the variance in the IBI. Further, we performed a blindfolding procedure (Ning and Liu, 2008) with an omission distance of seven to obtain the stone–Geisser’s *Q*^2^ value (Hair et al., 2014). The findings imply that the *Q*^2^ values for EOC, IBI, and ZYT were 0.495, 0.636, and 0.432, respectively. As both values were above zero, the research model was considered to have predictive relevance.

### Analysis of the Mediating Effect

We used bootstrapping and Sobel’s test to verify the effect Sobel (1982) test evaluates the significance of intermediaries by finding the product of coefficients (ACELS→EOC*EOC → IBI, etc.), which relies on the distribution assumption whereby the distribution of indirect effects tends to be asymmetric and skewed unless the mean is much greater than the SD (Stone and Sobel, 1990; MacKinnon et al., 2004). Since asymmetry affects the applicability of Sobel’s test when working with small sample sizes, Stine (1990) pioneered the deployment of bootstrapping to analyze mediation processes.

We estimated the path model using bootstrapping without the interaction of a mediator (Table 4). The outcomes reveal four direct paths that are statistically significant, which indicates that the mediation tests of ZYT and EOC are reasonable. We required the significance of indirect paths to verify that both EOC and ZYT mediate the relationship between ACELS and consumers’ IBI, and between SPELS and IBI. For H9b, the *t*-value of the indirect path (SPELS→EOC → IBI) is 3.147, with a value of *p* of 0.004. Hence, SPELS has a significant, indirect impact on IBI through EOC. Likewise, the *t*-value of the indirect path (SPELS→ZYT → IBI) was 5.198, i.e., significant at the 10% level. This shows that ZYT mediates the relationship between sales promotion and IBI, proving the H9a hypothesis. As for H8b, the *t*-value of the indirect path (ACELS→EOC → IBI) is 3.099, with a value of *p* of 0.003, i.e., significant at 10%, which demonstrates that the influence of ACELS on consumers’ IBI is mediated by EOC. Lastly, the *t*-value of 5.152 shows that ZYT mediates the relationship between ACELS and IBI, thereby establishing H8a (ACELS→ZYT → IBI).

**Table 4:** Test results summary (H1–H7).

Hypothesis	Relation	Path coefficient	Std. error	*T*	*p*	Significance	Tested results
H1	SPELS→ZYT	−0.524	0.060	8.702	<0.01	***	Supported
H2	SPELS→EOC	0.480	0.044	10.81	<0.01	***	Supported
H3	ACELS→ZYT	−0.321	0.049	6.512	<0.01	***	Supported
H4	ACELS→EOC	0.394	0.049	7.973	<0.01	***	Supported
H5	ZYT → IBI	−0.328	0.042	7.805	<0.01	***	Supported
H6	EOC → IBI	0.117	0.037	3.194	<0.01	***	Supported
H7	ZYT → EOC	−0.064	−0.065	0.061	0.295	ns	Not supported

***, *stands for the significance level of 0.001 and ns, refers to insignificance*.

According to Hair et al. (2014), the level of mediation is computed through the variance accounted for by VAF (indirect effect/total effect*100). As seen in Table 5, because the value of VAF is larger than 20 and smaller than 80, EOC partially mediates the relationship between ACELS and IBI. Likewise, ZYT partially mediates the relationship between SPELS, IBI, and ACELS. However, the mediating effect of EOC on SPELS and IBI is weak.

**Table 5:** Mediating test.

Effect	Path	Path coefficient	Indirect effect	SD	Total effect	VAF	*t*	*p*	Results
Direct without mediator	SPELS→IBI	0.620	Not applicable				13.24	<0.001	H9b supported
Indirect with mediator	SPELS→IBI	0.461	Not applicable		0.515	10.5%	3.147	0.004	
	SPELS→EOC	0.480	0.057	0.018					
	EOC→IBI	0.118							
Direct without mediator	SPELS→IBI	0.620	Not applicable				13.24	<0.001	H9a supported
Indirect with mediator	SPELS→IBI	0.461	Not applicable		0.635	27.4%	5.198	<0.001	
	SPELS→ZYT	−0.523	0.172	0.033					
	ZYT→IBI	−0.328							
Direct without mediator	ACELS→IBI	0.127	Not applicable				3.393	0.001	H8b supported
Indirect with mediator	ACELS→IBI	0.082	Not applicable		0.127	35.4%	3.099	0.003	
	ACELS→EOC	0.394	0.047	0.015					
	EOC→IBI	0.118							
Direct without mediator	ACELS→IBI	0.127	Not applicable				3.393	0.001	H8a supported
Indirect with mediator	ACELS→IBI	0.082	Not applicable		0.185	55.7%	5.152	<0.001	
	ACELS→ZYT	−0.319	0.103	0.020					
	ZYT→IBI	−0.323							

## Discussion and Conclusion

Although users’ perception of livestreaming has been discussed using flow theory (Oyedele and Simpson, 2018), self-identity theory (Yang and Lee, 2018), social identity theory (Hu et al., 2017), gratification theory (Mäntymäki and Islam, 2015), and perceived value theory (Rubio et al., 2019), it is still unclear how environmental stimuli of livestreaming play distinct roles in the inner organism of online consumers when they make impulsive buying decisions. Based on SOR theory, we examined the process of consumers’ IBI from external stimuli to internal organisms. Our findings are helpful for scholars and practitioners to thoroughly understand the psychology and behavior of livestreaming customers by emphasizing the formation of consumers’ impulsive purchase mechanisms during livestreaming. We incorporated a traditional Chinese cultural value, Zhong Yong, into the consumer behavior research model, which benefits multinational organizations and foreign brands in gaining insight into Chinese consumers’ preferences to utilize livestreaming marketing tools. Our empirical results provide valuable insight.

First, atmospheric cues and sales promotion in the livestreaming environment are two important factors in the mechanism of consumers’ impulsive buying. H4 shows that ACELS can directly influence EOC, which is in line with past research (e.g., Chen and Lin, 2018; Ma and Mei, 2018). In this study, we measured ACELS using three dimensions: the interactive, entertainment, and safety atmospheres. To provide alluring atmospheric cues, e-commerce livestreaming platforms should leverage information technology as an interactive medium and transmission bridge between livestreaming platforms, anchors, and customers. The “magic of bullet screen interactions” (Zhou et al., 2018), for example, provides a channel for online customers to ask questions spontaneously and to receive live-streamed comments from anchors, considerably improving information acquisition and marketing efficiency (Wongkitrungrueng et al., 2020). By employing a simple, easy-to-use, personalized, and amusing livestreaming environment to establish a comfortable and controllable communication channel, streamers can acquire competitiveness and generate a distinctive image in the community, which is the root of boosting sales, revenue, and profit.

According to the verification of H2 and H9b, promotion methods (i.e., time limits and material incentives) adopted for livestreaming will directly affect EOC and indirectly affect IBI by arousing emotions. Due to time-limited promotion, consumers feel pressed for time and become enthusiastic, prompting them to make rapid purchases. Anchors should employ “flash sale” promotions, which shorten the time it takes for consumers to make choices, reminding them of the urgency of time and letting them perceive time pressure. Owing to the significant growth in the number of visits and clicks during live promotions, emphasis should be placed on attentive customer service and reliable product quality assurance, rather than blindly focusing on front-end customer drainage. Furthermore, favorable challenges in the online shopping process, such as shopping and finding higher-quality products at low cost, might influence consumers’ emotional experiences (Wu et al., 2016). Hence, anchors should identify ideal material incentives for goods based on their product positioning on a live stream. For example, “price discounts” entice buyers to make spontaneous purchases when they are fully aware of a product’s characteristics. A “full cut” campaign is a price reduction that encourages customers to spend more money. Customers may be enticed to make a second purchase through the “full coupon” action, which is a kind of pricing promotion designed to encourage repeat purchases.

Second, according to the results regarding the organism, EOC and ZYT both influence impulsive buying in the livestreaming environment. In line with Youn and Faber (2000), the outcomes of H6 indicate that EOC has a positive impact on impulsive buying behavior. Some researchers have focused on this path but are mainly limited to traditional offline transactions (e.g., Weinberg and Gottwald, 1982; Piron, 1991). Thus, our findings add value to the livestreaming environment in China with genuine evidence, manifesting desired results that are helpful for practical implications; dynamic outcomes can also be acquired. As the organism of the SOR framework, EOC plays a mediating role in the path from the stimulus to the response, which is consistent with Donovan et al. (1994). To actively enhance consumers’ pleasure and emotions and provide them with lasting appeal, streamers should attach deep importance to consumers’ real-time interactive feedback and mobilize their sensory ability.

We incorporated a unique and novel mediator, Zhong Yong, to extensively explore the decision-making process of Chinese consumers in livestreaming. Zhong Yong philosophy represents a moral ideal in which a person’s affect, cognition, and behavior are constantly experienced and expressed in moderation: not too much or too little, but an appropriate balance (Ji and Chan, 2017). From the verification of hypotheses H8a and H9a, we empirically established the mediating role of ZYT in the relationship between the stimulus (i.e., ACELS and SPELS) and the response (i.e., IBI), which is in line with Qtaishat (2021). As the thinking mode casts light on a consumer’s behavior and predisposition before taking an action, analyzing the mediating role of ZYT provides a greater, more in-depth understanding of consumers’ purchase behavior. Our research partially fills this gap by positioning ZYT as a whole concept, including holistic orientation, perspective integration, and harmony maintenance, as well as expanding its real scenario to the livestreaming environment.

Chinese people with ZYT are more likely to obtain a holistic orientation, and to view products and promote them from global and comprehensive angles, weighing both the good and bad sides of their behavior on a cognitive basis (Wang et al., 2013). H5 suggests that ZYT dampens IBI, which is in line with Wu and Lin (2005). ZYT is key to the success of e-commerce marketing to ensure that sales promotion makes it profitable to sell to consumers and that messages are accurately transmitted to them through effective channels. Apart from strengthening publicity, merchants should pay attention to promotion strategies and the diversification of promotion channels. The forecast of livestreaming promotion is often displayed a few days before a broadcast, and consumers can fully grasp product attributes such as brand, size, and usage scenario in advance, reducing the time and energy needed to look for information. When it comes to a broadcast, detailed and patient explanations by the anchor, together with huge price discounts or other generous material incentives, will greatly arouse consumers’ instant desire to make a purchase.

Perspective integration requires individuals to integrate perspectives on all sides and is a process of self-reflection and self-control. Consumers with perspective integration tend to behave according to the cognitive processing system rather than the emotional processing system, thus restraining IBI. E-commerce businesses should try to understand Chinese consumers’ personality traits and habits to avoid their antagonistic psychology caused by excessive marketing. The threshold price should not be set too high for a “full cut” promotion, and the magnitude of a sales discount should not be excessive. Otherwise, consumers with ZYT will doubt brand integrity and product value, thus discouraging purchase tendency or even giving up buying the products in the end. Merchants should consider the characteristics of Chinese consumers and choose marketing strategies suitable for the Chinese public. To prove it with a vivid example, compared to “maximum 1,000 minuses 200,” Chinese consumers are more likely to accept “maximum 300 minus 60.”

Finally, people with ZYT aim to maintain harmony. Unlike the Western cultural values of rivalry and control, Confucian Zhong Yong seeks harmony between people and nature. Based on the awe of heaven, it is emphasized that the gift of nature should be exploited responsibly. Chinese citizens traditionally reject consumerism since it lacks a clear, deliberate attitude on the central point of maintaining the human-nature balance; they leap out of materialism, limiting the desire for prosperity. As a result, focusing too much on passenger flow and gross merchandise volume while ignoring brand building and operational efficiency will increase consumer concerns and conservatism, and will further deteriorate the marketing environment and competitive advantage for enterprises.

ZYT is a cognitive factor that constitutes an organism, along with EOC. Although many studies have shown that Zhong Yong exerts emotional control (Ji et al., 2010), the effect of ZYT on EOC is not significant; that is, H7 does not hold. Based on the affective-cognitive mode, we propose that the emotional and cognitive systems function independently and in parallel, which is in line with Wang et al. (2015). During the decision-making process, the processing directions of the two systems may be consistent or opposite. In the impulsive buying scenario, consumers choose between two paths through the ZYT or EOC, which lead in opposite directions. The final response is stimulated by dominance. In sum, we explored a unique way to explain impulsive buying behavior in livestreaming from the standpoint of the traditional Chinese value of Zhong Yong. However, we suggest that the effect of ZYT on consumer behavior may be far more complex, thus warranting a more comprehensive investigation in future.

## Data Availability Statement

The raw data supporting the conclusions of this article will be made available by the authors, without undue reservation.

## Ethics Statement

Ethical review and approval was not required for the study on human participants in accordance with the local legislation and institutional requirements. Written informed consent from the (patients/participants or patients/participants legal guardian/next of kin) was not required to participate in this study in accordance with the national legislation and the institutional requirements.

## Author Contributions

HongliG is responsible for model building, data analysis, and paper writing. XC is responsible for concept determination or design and thesis writing. HonglinG is responsible for data collection and collation. BY is in charge of project guidance and writing coordination. All authors contributed to the article and approved the submitted version.

## Conflict of Interest

The authors declare that the research was conducted in the absence of any commercial or financial relationships that could be construed as a potential conflict of interest.

## Publisher’s Note

All claims expressed in this article are solely those of the authors and do not necessarily represent those of their affiliated organizations, or those of the publisher, the editors and the reviewers. Any product that may be evaluated in this article, or claim that may be made by its manufacturer, is not guaranteed or endorsed by the publisher.

## Figures and Tables

**Table:** 

Construct	Items#	Scale items	References
Emotion of online consumers (EOC)	EOC1	Pleased-annoyed	Mehrabian and Russell, 1974; Cheng et al., 2009
	EOC2	Satisfied	
	EOC3	Stimulated	
	EOC4	Excited	
Zhong Yong tendency (ZYT)	ZYT1	I maintain the right balance, do not go to extremes, and consider the opinions of others.	Wu and Lin, 2005; Zhou and Yang, 2021
	ZYT2	I try to find a balance between my own opinions and those of others.	
	ZYT3	When making decisions, I usually adjust my expressions for the harmony of the whole.	
	ZYT4	When there is an argument, I will try to find a balance between opinions of mine and others.	
	ZYT5	I try to maintain harmony in all things and not to conflict with others as much as possible.	
	ZYT6	Good interpersonal relationships are more important to me than my own achievements.	
Sales promotion on e-commerce livestreaming (SPELS)	SPELS1	I feel that the countdown promotion time specified in the livestreaming is relatively short.	Rook, 1987; Duncan Herrington and Capella, 1995; Lin and Lin, 2013; Chou, 2019
	SPELS2	Before the deadline of sales promotion on livestreaming, there is very little time left for making the decision.	
	SPELS3	The closer to the end of the time-limited sale promotion, the more time pressure will push me to buy as soon as possible.	
	SPELS4	I will keep watching the live streaming to receive coupons and freebies or to enter a prize draw.	
Atmospheric cues in the e-commerce livestreaming (ACELS)	ACELS1	I can obtain other customers’ opinions and questions from the bullet screen, which is supplementary information for my decision.	Dmitri, 2017; Chen and Lin, 2018; Wongkitrungrueng and Assarut, 2020; Wang et al., 2022
	ACELS2	I participate in the livestreaming activities such as “liked,” gifts and lottery.	
	ACELS3	The e-commerce livestreaming content made me feel intrigued and at ease.	
	ACELS4	I feel delighted and content when watching e-commerce livestreaming.	
	ACELS5	The presentation and description of goods in the livestreaming are almost always accurate.	
Impulsive buying intention (IBI)	IBI1	I buy things recommended by the anchor without careful consideration.	Beatty and Ferrell, 1998; Liu et al., 2013; Dmitri, 2017
	IBI2	I often have a sudden and strong desire to buy in the livestreaming.	
	IBI3	I will be unsatisfied if I do not buy something I like in the e-commerce livestreaming.	
	IBI4	I buy things that I have not intended to purchase in the e-commerce livestreaming.	

## Appendix

Constructs and items.
