# Corrigendum: Understanding Chinese consumers' livestreaming impulsive buying: An stimulus-organism-response perspective and the mediating role of emotions and Zhong Yong tendency

**DOI:** 10.3389/fpsyg.2023.1138831

**Published:** 2023-02-23

**Authors:** Hongli Gao, Xinzhi Chen, Hongling Gao, Bin Yu

**Affiliations:** ^1^School of Economics, Zhejiang University of Technology, Hangzhou, China; ^2^School of Economics and Management, Zhejiang University of Science and Technology, Hangzhou, China

**Keywords:** impulsive buying, SOR theory, Zhong Yong tendency, livestreaming, atmospheric cues

In the published article, there was an error. The Funding statement was excluded from the published article. The correct Funding statement appears below.

